# Leg Lengthening as a Means of Improving Ambulation Following an Internal Hemipelvectomy

**DOI:** 10.1155/2016/7089142

**Published:** 2016-10-09

**Authors:** Wakyo Sato, Hiroshi Okazaki, Takahiro Goto

**Affiliations:** ^1^Department of Rehabilitation Medicine, Tokyo Metropolitan Tama Medical Center, 2-8-29 Musashidai, Fuchu-Shi, Tokyo 183-8524, Japan; ^2^Department of Orthopedic Surgery, Japan Labour Health and Welfare Organization, Kanto Rosai Hospital, 1-1 Kizukisumiyoshi-cho, Nakahara-ku, Kawasaki City, Kanagawa 211-8510, Japan; ^3^Department of Musculoskeletal Oncology, Tokyo Metropolitan Cancer and Infectious Diseases Center Komagome Hospital, 3-18-22 Honkomagome, Bunkyo-ku, Tokyo 113-8677, Japan

## Abstract

Reconstructive surgery following an internal hemipelvectomy for a malignant pelvic tumor is difficult due to the structural complexity of the pelvis and the massive extension of the tumor. While high complication rates have been encountered in various types of reconstructive surgery, resection without reconstruction reportedly involved fewer complications. However, this method often results in limb shortening with resultant instability during walking. We reported herein leg lengthening performed to correct lower limb shortening after an internal hemipelvectomy, which improved ambulatory stability and overall QOL. An 18-year-old male patient came to our hospital to correct a lower limb discrepancy resulting from a left internal hemipelvectomy. His left pelvis and proximal femur had been resected, and the femur remained without an acetabular roof. His left lower limb was about 8 centimeters shorter. The left tibia was lengthened 8 centimeters with an external fixator. After the lengthening, the patient was able to walk without support and his gait remarkably improved. Additionally he no longer required placing a wallet in his back pocket as a pad as a means of raising the left side of his torso while sitting. Leg lengthening was a useful method of improving ambulation after an internal hemipelvectomy.

## 1. Introduction

The treatment of pelvic tumors involving the acetabulum in particular is difficult due to the anatomical complexity of the pelvis and the massive extension of the tumor [1]. Ablation of the affected lower limb was a standard procedure in the past, but, as medicine advanced, limb-sparing procedures such as the internal hemipelvectomy have become popular [2]. Reconstructing the pelvis after an internal hemipelvectomy has nonetheless remained difficult. Prosthetic implantation [3, 4], bone graft, bone cement reconstruction, arthrodesis, hip transposition, or a combination of some of these methods has been employed [4, 5]. Although reconstructive surgery is preferable to amputation, a complication rate as high as 50–60% has been cited in connection with this method [3, 6–10].

Resection without reconstruction, that is, the “flail hip,” is an alternative form of pelvic tumor treatment [11, 12] but the result was reportedly poor in terms of restoration of function [6, 13]. Other, more recent reports, on the other hand, demonstrated a lower complication rate and higher functional score for this procedure [8, 12] in which the shortening of the lower limb resulted in a gait abnormality. Rectifying the discrepancy in length has reportedly improved lower extremity stability and ambulation [12, 14].

The purpose of this paper is to assess bone lengthening as a method of correcting leg length discrepancy and improving ambulation following an internal hemipelvectomy, especially in cases involving a nearly complete resection of the half pelvis and the ipsilateral proximal femur.

## 2. Case Presentation

A 13-year-old boy received the diagnosis of clear cell chondrosarcoma in his left ilium and underwent a left internal hemipelvectomy. The whole left ilium, most of the left ischium, part of superior pubis ramus, and the left femoral head and neck were resected. The total iliacus and the psoas major muscle were resected while the tendon of the psoas muscle was left intact. About half of the left gluteus maximus and medius muscles were left and resutured to the abdomen or back muscles after bone resection, preserving the extension and abduction functions of the left hip joint. The left adductor muscles were left intact.

**Figure 1:**
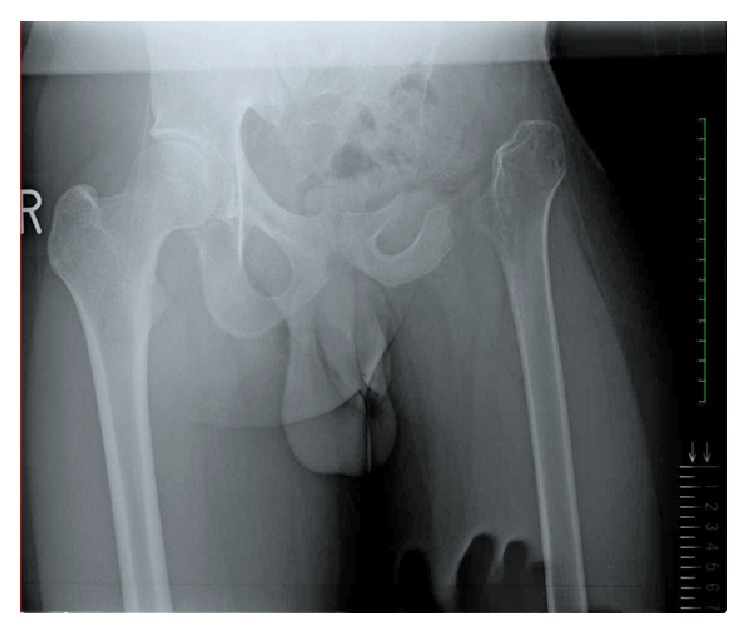
Radiograph of the hip joints at first visit.

**Figure 2:**
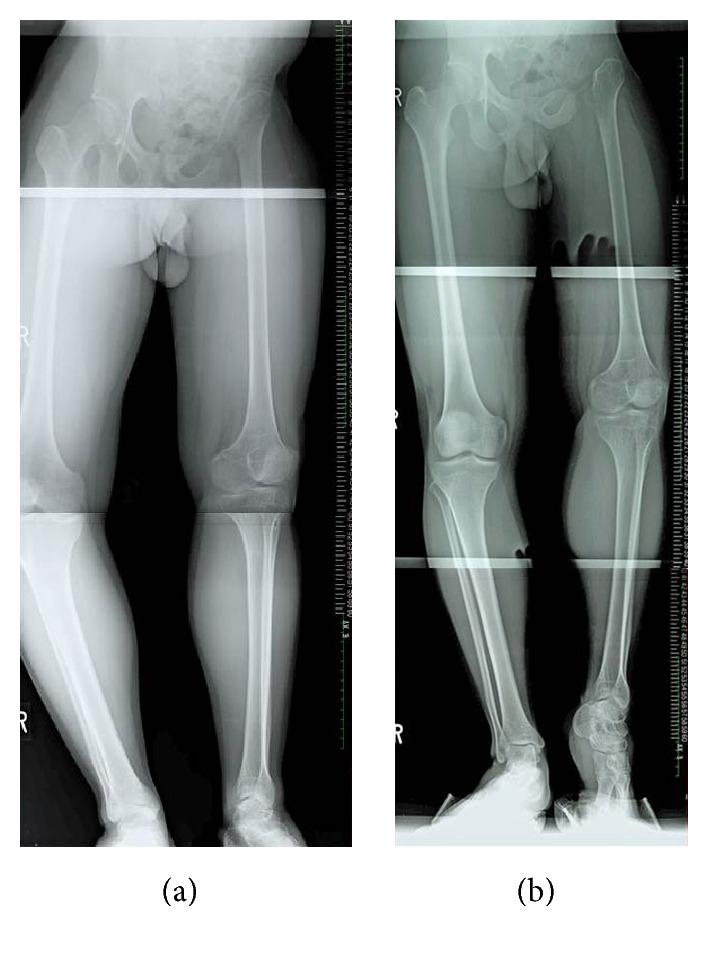
Radiographs of entire lower limbs before leg lengthening showed tip of the residual left femur had moved upward about 4 cm in standing position (a) compared with the right leg (b).

At 18 years old, the patient came to our hospital to correct the discrepancy in the length of his legs. A hip radiograph showed evidence of surgical resection of the left pelvis, femoral head, and neck (Figure 1). A radiograph of the entire lower limb revealed that the tip of the residual left femur moved upward about 4 centimeters on the left leg compared with the right leg in the standing position (Figure 2). The patient walked with a limp and required a crutch. The umbilicus-medial malleolus distance (UMD) was 87 cm/82 cm (right/left) when in the standing position on the right leg and 87 cm/77 cm when on the left leg.

**Figure 3:**
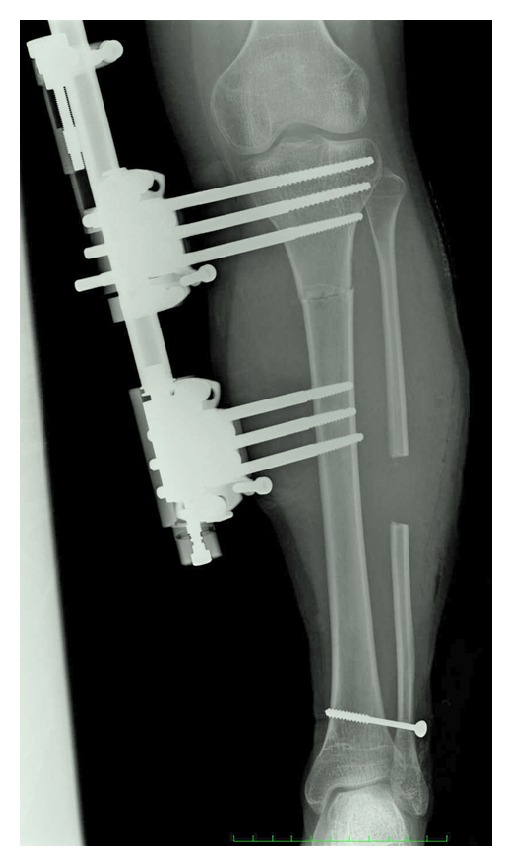
Radiograph of the left tibia after lengthening operation.

Before the operation to lengthen the limb, we gauged the desired effect using a shoe-lift to simulate changes in limb length. After several trials, an 8-centimeter shoe-lift raised the pelvis sufficiently to achieve evenness in the height and position of the hips. Then, the left tibia was lengthened using an unilateral external fixator (Hifixator®, Nagano Keiki Co. Ltd., Ueda, Japan). The osteotomy was performed at the proximal tibia, and three half pins 6 mm in diameter were inserted above and below the osteotomy site (Figure 3). The patient began walking with crutches on the day after the operation. Weight-bearing was strongly encouraged. Lengthening began at one week after the operation at an average rate of 0.5 mm/day. The total increase in length was 8 cm, and the duration of fixator mount use was 17.5 months. A below-the-knee cylinder brace was worn when walking for 9 months after fixator removal.

**Figure 4:**
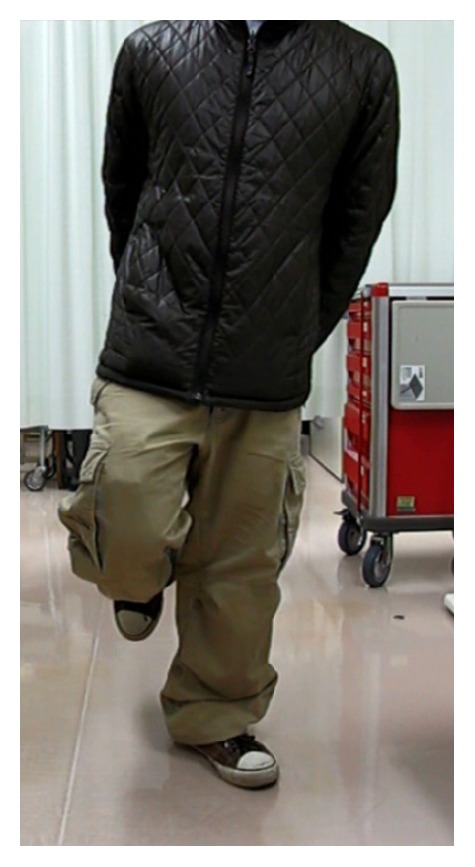
Photograph showed he stood on the left leg steadily without any support.

**Figure 5:**
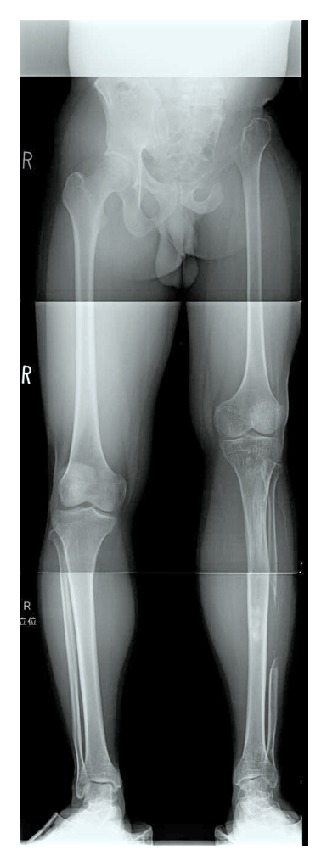
Radiograph of entire lower limbs at 5 years after leg lengthening.

The patient was able to walk short distances without a crutch after fixator removal. One year later, he was able to walk without any assistance. Five years after fixator removal, he was able to walk 4-5 kilometers continuously without any apparent gait abnormality and could stand on the left leg steadily (Figure 4). The radiograph of the entire lower limbs in the standing position showed the tip of the left femur in the same position as when the 8 cm shoe-lift was used (Figure 5). Furthermore, after lengthening, the patient no longer required placing a wallet in his back pocket to adjust his height in the seated position. The patient expressed great satisfaction with the results. The Musculoskeletal Tumor Society Score (MSTS) [15] was 26 (87%).

## 3. Discussion

The annual incidence of bone malignancies is about 4–7 cases/million children under 15 years of age [16, 17]. Nearly two-thirds of the malignancies occur in the pelvis [18].

While nonreconstruction following an hemipelvectomy can prevent complications such as those that occur when reconstruction is performed, some gait impairment will result from the discrepancy in limb length and the resultant instability of the lower limb.

As a solution to this problem, Eilber et al. [14] reported that a shoe-lift or compensatory pelvic tilt was effective in rectifying an average discrepancy of 3.5 cm. Schwartz et al. [12] reported that a shoe-lift compensating for approximately 50% of the total discrepancy improved stability and ambulation. Catagani and Ottaviani [19] reported favorable results with an increase of 6.4 cm in leg length using an Ilizarov apparatus.

In the present case, the discrepancy was approximately 8 centimeters although some variation resulted from the difference in loading on the affected and healthy limb. The 8 cm shoe-lift was effective, but in addition to being uncomfortably heavy, this height also made the patient prone to tripping over and too heavy to walk. Nonetheless the surgical lengthening was generally very favorable, allowing the patient not only to walk without any ambulatory aids but also to sit on a chair without using a wallet to bolster his torso.

Another concern following the procedure was the possibility of the tip of the residual femur penetrating overlying soft tissue by loading during ambulation. In a study of the course of 98 untreated cases of congenital hip dislocation (CHD) among patients aged 17–65 involving a total of 132 hips, Kono [20] reported that 68 patients suffered complete dislocation of the hip. In 64 of these cases, the femoral head was situated in the gluteus muscle without the acetabulum but was able to maintain stability and mobility during the patients' lifetime. The features of our case resembled those of a complete CHD with an unstable lower limb. Although our patient suffered from a gait abnormality like that of the CHD patients, surgical intervention restored lower limb stability and enabled him to walk without any ambulatory aids.

To assess the functional efficacy of flail hips, Takami et al. [21] reviewed 5 cases with MSTS scores between 53% and 93%. Schwartz et al. [12] reported 8 nonreconstruction cases with MSTS scores at 53.3–80.0% of normal values. These reports demonstrated that nonreconstruction methods are capable of producing acceptable outcomes. Our case also demonstrated a favorable result with a MSTS score of 87%.

The present report features only one case of surgical intervention to treat the shortening of a lower limb due to an hemipelvectomy. The result was nonetheless very favorable from both the clinical perspective and patient satisfaction. We consider this method as a viable option for the treatment of impaired ambulation due to an hemipelvectomy.

## 4. Conclusion

Lengthening the shortened limb was a useful method of improving ambulation following an internal hemipelvectomy.

## Supplementary Material

Five years after fixator removal, the patient walked without apparent gait abnormality, and could stand on the lengthened leg steadily without any support.

## References

[1] Veth R. P. H., van Hoesel R., Pruszczynski M., Hoogenhout J., Schreuder B., Wobbes T. (2003). Limb salvage in musculoskeletal oncology. *The Lancet Oncology*.

[2] Nagarajan R., Neglia J. P., Clohisy D. R., Robison L. L. (2002). Limb salvage and amputation in survivors of pediatric lower-extremity bone tumors: what are the long-term implications?. *Journal of Clinical Oncology*.

[3] Abudu A., Grimer R. J., Cannon S. R., Carter S. R., Sneath R. S. (1997). Reconstruction of the hemipelvis after the excision of malignant tumours. *The Journal of Bone and Joint Surgery—British Volume*.

[4] Satcher R. L., O'Donnell R. J., Johnston J. O. (2003). Reconstruction of the pelvis after resection of tumors about the acetabulum. *Clinical Orthopaedics and Related Research*.

[5] Schwameis E., Dominkus M., Krepler P. (2002). Reconstruction of the pelvis after tumor resection in children and adolescents. *Clinical Orthopaedics and Related Research*.

[6] Windhager R., Karner J., Kutschera H.-P., Polterauer P., Salzer-Kuntschik M., Kotz R. (1996). Limb salvage in periacetabular sarcomas: review of 21 consecutive cases. *Clinical Orthopaedics and Related Research*.

[7] Renard A. J. S., Veth R. P. H., Schreuder H. W. B. (2000). The saddle prosthesis in pelvic primary and secondary musculoskeletal tumors: functional results at several postoperative intervals. *Archives of Orthopaedic and Trauma Surgery*.

[8] Hillmann A., Hoffman C., Gosheger G., Rödl R., Winkelmann W., Ozaki T. (2003). Tumors of the pelvis: complications after reconstruction. *Archives of Orthopaedic and Trauma Surgery*.

[9] Menendez L. R., Ahlmann E. R., Falkinstein Y., Allison D. C. (2009). Periacetabular reconstruction with a new endoprosthesis. *Clinical Orthopaedics and Related Research*.

[10] Aydinli U., Ozturk C., Yalcinkaya U., Tirelioglu O., Ersozlu S. (2004). Limb-sparing surgery for primary malignant tumours of the pelvis. *Acta Orthopaedica Belgica*.

[11] Steel H. H. (1978). Partial or complete resection of the hemipelvis. An alternative to hindquarter amputation for periacetabular chondrosarcoma of the pelvis. *The Journal of Bone and Joint Surgery—American Volume*.

[12] Schwartz A. J., Kiatisevi P., Eilber F. C., Eilber F. R., Eckardt J. J. (2009). The friedman-eilber resection arthroplasty of the pelvis. *Clinical Orthopaedics and Related Research*.

[13] O'Connor M. I. (1997). Malignant pelvic tumors: limb-sparing resection and reconstruction. *Seminars in Surgical Oncology*.

[14] Eilber F. R., Grant T. T., Sakai D., Morton D. L. (1979). Internal hemipelvectomy—excision of the hemipelvis with limb preservation. An alternative to hemipelvectomy. *Cancer*.

[15] Enneking W. F., Dunham W., Gebhardt M. C., Malawar M., Pritchard D. J. (1993). A system for the functional evaluation of reconstructive procedures after surgical treatment of tumors of the musculoskeletal system. *Clinical Orthopaedics and Related Research*.

[16] Arndt C. A., Crist W. M. (1999). Common musculoskeletal tumors of childhood and adolescence. *The New England Journal of Medicine*.

[17] Marugame T., Katanoda K., Matsuda T. (2007). The Japan cancer surveillance report: incidence of childhood, bone, penis and testis cancers. *Japanese Journal of Clinical Oncology*.

[18] Nagarajan R., Clohisy D. R., Neglia J. P. (2004). Function and quality-of-life of survivors of pelvic and lower extremity osteosarcoma and Ewing's sarcoma: the Childhood Cancer Survivor Study. *British Journal of Cancer*.

[19] Catagani M. A., Ottaviani G. (2008). Ilizarov method to correct limb length discrepancy after limb-sparing hemipelvectomy. *Journal of Pediatric Orthopaedics Part B*.

[20] Kono S. (1967). Untreated severe congenital hip dislocations. *Journal of the Japanese Orthopaedic Association*.

[21] Takami M., Ieguchi M., Takamatsu K. (1997). Functional evaluation of flail hip joint after periacetabular resection of the pelvis. *Osaka City Medical Journal*.

